# Mycotoxin profiles of animal feeds in the central part of Thailand: 2015-2020

**DOI:** 10.14202/vetworld.2021.739-743

**Published:** 2021-03-23

**Authors:** Suppada Kananub, Prakorn Jala, Sudtisa Laopiem, Alongkot Boonsoongnern, Arsooth Sanguankiat

**Affiliations:** 1 Department of Veterinary Public Health, Faculty of Veterinary Medicine, Kasetsart University, Kamphaeng Saen, Nakhon Pathom, Thailand; 2 Kamphaeng Saen Veterinary Diagnostic Center, Faculty of Veterinary Medicine, Kasetsart University, Kamphaeng Saen, Nakhon Pathom, Thailand; 3 Department of Farm Resources and Production Medicine, Faculty of Veterinary Medicine, Kasetsart University, Kamphaeng Saen, Nakhon Pathom, Thailand

**Keywords:** complete feeds, compound feeds, contamination, enzyme-linked immunosorbent assay, mycotoxin

## Abstract

**Background and Aim:**

Mycotoxin contamination in animal feeds is of considerable concern because it can affect animal health systems. As a result of contamination in the food chain, humans can indirectly come into contact with mycotoxins. The present study aimed to present mycotoxin contamination patterns in animal feeds from 2015 to 2020 and elucidate associations between the type of feed and the type of ingredient.

**Materials and Methods:**

Data were summarized from the records of the Kamphaeng Saen Veterinary Diagnosis Center from 2015 to 2020, which comprised the analyses of aflatoxin (AFL), zearalenone (ZEA), T-2 toxin (T-2), fumonisin (FUM), and deoxynivalenol (DON) contamination in feed ingredients, complete feeds, and unclassified feeds. Descriptive statistics, Chi-squared tests, and Fisher’s exact tests were used for data analysis.

**Results:**

ZEA was prevalent in animal feeds. The prevalence of each mycotoxin was constant from 2015 to 2020. Approximately 20-30% of samples were positive for AFL and FUM. The highest contamination was ZEA, which was found in 50% of the samples, and the occurrence of T-2 and DON was <10%. AFL significantly contaminated complete feeds more than feed ingredients. Feed ingredients were related to mycotoxin contaminations. The highest levels of AFL, FUM, and DON contamination occurred in 2017. The data in this year consisted mostly of soybean, corn, and rice bran.

**Conclusion:**

The number of positive samples of all five mycotoxins was constant from 2015 to 2020, but the occurrence of ZEA was the highest. Mycotoxins in feedstuffs are significantly related to the type of feed and the type of ingredient.

## Introduction

Animal feeds and forages contain a wide range of contaminants and toxins. Contamination with mycotoxins, which are secondary metabolites produced by fungi present in forages, cereals, and compound feeds of livestock, is a global issue. Fungal contamination affects animal health, and hence the animal industry, by influencing the nutritional value and palatability of feed, and causes mycotoxicosis in animal [1-3]. The most economically important mycotoxins in terms of their prevalence and undesirable effects on animal performance are aflatoxin B1, deoxynivalenol (DON), zearalenone (ZEA), ochratoxin A, trichothecenes, and fumonisin B1 [4]. These mycotoxins are produced mainly by *Aspergillus*, *Fusarium*, and *Penicillium*, the primary fungi related to the contamination of food and animal feeds [5-7].

As a result of contamination in the food chain, humans can indirectly contact the mycotoxin [5,7,8]. An adverse effect of consumption of toxins such as aflatoxin (AFL) is liver cancer in humans; it has also been associated with stunting and other health problems. The consumption of AFLs in high amounts by animals also results in severe, sudden onset of illness, and death [9]. There is evidence of chemical and biological alterations of mycotoxins by thermal modifications during processing; fungus-, plant-, or animal-derived metabolites of matrix-associated mycotoxins have increasingly been recorded in recent years, and they may contribute to overall mycotoxin exposure [10]. Several factors influence the growth of fungi and alter the nutritional requirements of animals, such as species, breed, sex, ration consumption, diet energy level, nutrient availability, temperature, air humidity, and animal health status. The occurrence of mycotoxins in each area might be different [1,2,5,11]. The profiles of mycotoxins in animal feeds are essential for the manufacturers to improve animal performance and products [12].

The aim of this study was to determine the mycotoxin profiles of animal feeds in Thailand, with data derived from a veterinary diagnostic laboratory in West Thailand from 2015 to 2020, and to elucidate the associations between the type of feed and type of ingredient.

## Materials and Methods

### Ethical approval

Ethical approval was not required for this study.

### Data collection

The data in this study were summarized from records of the Kamphaeng Saen Veterinary Diagnostic Center from 2015 to 2020. All samples were analyzed by enzyme-linked immunosorbent assay (ELISA) from Romer^®^ Labs (Getzersdorf, Austria), and Neogen^®^ Corporation (Lansing, Michigan, USA), according to the manufacturer’s instructions. AgraQuant^®^ AFL, AgraQuant^®^ ZEA Plus, and AgraQuant^®^ Fumonisin (FUM) ELISA tests by Romer^®^ Labs were used to quantify the total amount of AFL, ZEA, and FUM; the ranges of detection were 4-40 ppb, 25-1000 ppb, and 250-5000 ppb, respectively. Veratox^®^ T-s/HT-2 Toxin (T-2) and Veratox^®^ Don 2/3 from Neogen^®^ Corporation were used to quantify the amount of T-2 and DON, with detection limits of 25-250 ppb and 500-6000 ppb, respectively.

### Statistical analysis

The prevalence of mycotoxin contamination according to sampling year, type of sample, and type of feed ingredient was tabulated. Feed ingredients, complete feeds, and unclassified feeds were compared in this study. Ingredients such as rice bran, soybean, cassava chips, corn, rice, and unclassified ingredients were categorized by mycotoxin levels according to the permitted levels recommended in the previous studies [1,13,14]. Samples were identified as CS: Cassava chip, RI: Rice, RB: Rice bran, CO: Corn, and SB: Soybean, while other unidentified feed samples were called “other” for which identification was not available. From these studies, permitted levels in agricultural products are 20 ppb of AFL, 100 ppb of ZEA, 250 ppb of T-2, 5000 ppb of FUM, and 900 ppb of DON. The independence between the appearance of mycotoxin and other factors groups was assessed using Pearson’s Chi-squared test and Fisher’s exact test by STATA software version 13.1 (StataCorp, College Station, TX, USA) [15]. p<0.05 was considered statistically significant.

## Results

This study included 3852 data entries from 2015 to 2020. The occurrence of mycotoxins and the level of detection in each year are shown in Table-1. The highest sample (893 records) was in 2015, while the lowest sample (243 records) was found in 2020 (with only 6 months data). Considering the mycotoxin tests performed during 2015 and 2020, the highest demand for testing was ZEA followed by AFL and FUM. Middle to low levels of contamination were found for all toxins. High-level contamination was found for AFL (2.3%), while T-2 did not feature in high-level contaminations. Half of the ZEA samples (54.1%) contained 25-1000 ppb. Low range contaminations of AFL, T-2, FUM, and DON were found in 71.7%, 94.7%, 72%, and 92.8% of samples, respectively (Table-1).

**Table-1:** AFL, ZEA, T-2, FUM, and DON contamination in feed samples in the central part of Thailand from 2015 to 2020.

Mycotoxin	Total	Range	No. of samples						%

			2015 (n^1^=893)	2016 (n=863)	2017 (n=841)	2018 (n=553)	2019 (n=459)	2020 (Jan. to June) (n=243)	
AFL (ppb)	2564	<4	404	431	401	258	223	121	71.68
		4-40	206	125	124	89	88	35	26.01
		>40	16	11	7	13	8	4	2.30
Zearalenone (ppb)	2699	<25	266	251	267	150	178	126	45.87
		25-1000	330	410	332	195	159	34	54.09
		>1000	0	1	0	0	0	0	0.04
T-2 (ppb)	1829	<25	372	432	404	225	199	100	94.70
		25-250	28	31	31	6	1	0	5.30
		>250	0	0	0	0	0	0	0.00
FUM (ppm)	2294	<0.25	330	375	371	283	234	68	72.41
		0.25-5	108	146	145	89	42	68	26.07
		>5	4	6	12	6	5	2	1.53
DON (ppm)	719	<0.5	150	98	166	107	102	44	92.77
		0.5-6	18	15	13	3	0	2	7.09
		>6	0	0	1	0	0	0	0.14

1 N=The total number of samples each year. AFL=Aflatoxin, ZEA=Zearalenone, T-2=T-2 toxin, FUM=Fumonisin, DON=Deoxynivalenol

**Figure-1:**
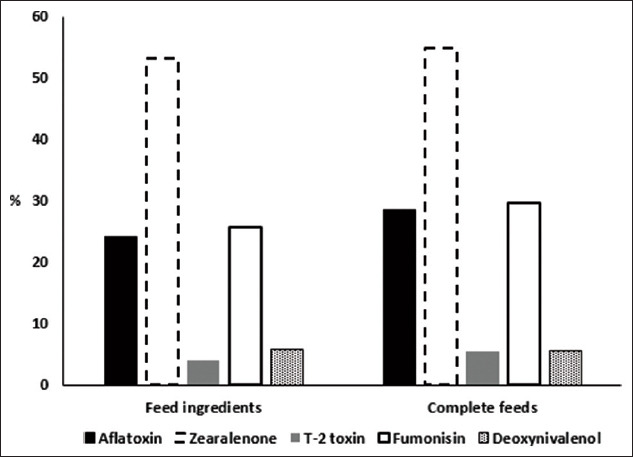
Prevalence of mycotoxin contamination in feed ingredients and complete feeds in the central part of Thailand from 2015 to 2020.

Table-2 presents mycotoxin levels based on samples in the detection range. Overall, the median of each toxin did not deviate in each year. The greatest variation was observed in FUM, for which the highest level was twice as likely as the lowest level. There were 2512 and 932 feed ingredients and complete feeds, respectively, whereas the unclassified group comprised 408 samples. The current study excluded the unclassified group because the type of feed could not be identified. The existence of AFL depended on the type of feed (p<0.05). The incidence of AFL contamination in complete feeds was 5% higher than that in feed ingredients (Figure-1).

**Table-2:** Mycotoxin levels based on the samples in the detection ranges in the central part of Thailand from 2015 to 2020.

Mycotoxin	Median IQR^1^ (min–max)					

	2015	2016	2017	2018	2019	2020 (Jan. to June)
AFL (ppb)	6.10-5 (4-39.70)	5.80-4.40 (4-38.20)	6.60-7.85 (4-37.70)	5.50-3.80 (4-38.70)	5.30-3.40 (4-33.60)	5.50-4.10 (4-32.50)
ZEA (ppb)	49.20-49 (25.10-477.50)	43.10-35.20 (25.10-459.8)	44.20-38.60 (25-598.90)	39.3-27.8 (25.10-879.60)	40.80-28.20 (25-324.80)	36.50-15.10 (25.10-124.40)
T-2 (ppb)	29.60-8.10 (25.10-64.10)	35.60-15.90 (25.30-65.10)	33-11.70 (25.40-112)	28.65-4.40 (25.30-33.90)	N/A^2^	N/A
FUM (ppm)	0.62-0.73 (0.25-4.07)	1.01-1.55 (0.25-4.72)	1.17-1.59 (0.25-4.85)	1.10-1.22 (0.25-4.49)	0.65-0.73 (0.25-3.39)	0.85-1.01 (0.26-3.81)
DON (ppm)	0.60-0.20 (0.50-3.10)	0.60-0.40 (0.50-1.30)	0.70-0.20 (0.50-1)	0.50-0.20 (0.50-0.70)	N/A	0.55-0.10 (0.50-0.60)

1 IQR=Interquartile range,

2 N/A=No observation or only one observation, AFL=Aflatoxin, ZEA=Zearalenone,T-2=T-2 toxin, FUM=Fumonisin, DON=Deoxynivalenol

**Figure-2:**
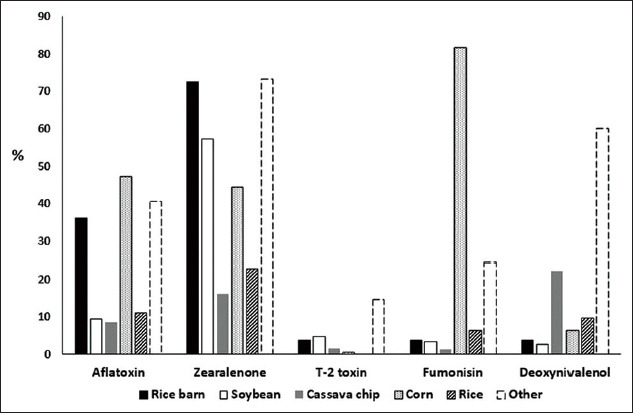
Percentage contamination as defined by feed ingredients (CS=Cassava chip, RI=Rice, RB=Rice bran, CO=Corn, SB=Soybean, Other=Ingredient identity not available).

The relationship between the contamination and the type of ingredient was significant (p<0.05). AFL was highly positive in corn and rice bran and “unclassified ingredient” samples. The ZEA contamination was higher than 60% in rice bran, soybean, and “unclassified ingredient” samples. About 15% of unclassified feed was contaminated with T-2 and <6% of the rest of the feed ingredients were contaminated with T-2. The highest occurrence of DON was in “unclassified ingredient” samples, which was 3 times higher than the contamination in cassava chips. FUM contaminated corn in up to 80% of samples (Figure-2). Less than 5% of feed ingredients and complete feeds were higher than the allowable levels in agricultural products; therefore, their relationship with other factors was not analyzed.

## Discussion

The occurrence of mycotoxins in this study did not conform to other studies [5,12,16]. The mycotoxin most prevalent in this study was ZEA, which concurred with D’Mello [11]. Rodrigues *et al*. [14] indicated that the occurrence of FUM and type B trichothecenes was highest. A study in Thailand showed 100% T-2 ­contamination in feed ingredients, but there was none in the Middle East and Africa, whereas the contamination in this study was approximately 10%.

The prevalence of AFL and DON was higher than this study in the work of Charoenpornsook and Kavisarasai [6], Rodrigues *et al*. [14]. The T-2 contamination in the current study was similar to that of DON, but Cegielska-Radziejewska *et al*. [12] showed that the prevalence of DON was 5 times higher than that of T-2. DON and FUM contaminations in the samples were 59% and 64%, respectively, in the study of Rodrigues and Naehrer [16] which differed from the current study. Contamination varied greatly between the studies since the existence of mycotoxins depends primarily on the study area [14]. Temperature and humidity are major causes for the difference in contamination incidence in each area and each year [17,18]. Appropriate storage conditions could prevent an increase in mycotoxin production [19].

The limits of detection reported by Charoenpornsook and Kavisarasai [6] based on their study in Thailand, were less than those in the current study, considering the high prevalence of AFL and DON, and 100% T2 contamination. Considering the occurrence of toxins in samples over the ­allowable limits, the incidence of contamination in the current study was lower than that in the study of Jala *et al*. [20], from the same area. However, our result is consistent with this study, that is, complete feeds were more contaminated than their ingredients.

The median of mycotoxin contamination throughout the study period was consistent. However, certain mycotoxins (AFL, FUM, and DON) were high in 2017. The contamination of AFL in the current study was related to the type of feed. Weather commonly influences the occurrence of mycotoxins in feedstuff; therefore, the kind of mycotoxin differed with time and area. The occurrence deviates from area to area or year to year [3,5,12,16]. Mold growth is chiefly related to temperature and humidity. Dry conditions increase the stress, leading to a decrease in plant immunity [5]. As a consequence of low immunity, crops could be sensitive to mycotoxin growth. Precipitation also stimulates the opportunity of infection by microorganisms [1,5]. Southeast Asia is, therefore, an area that has a high prevalence of AFL [3,14].

Significant contamination of feed ingredients was similar to that reported in other studies [13,21,22]. Contamination with AFL, ZEA, and DON frequently occurs in feed ingredients. The most severe contaminant is DON followed by ZEA and AFL [13,21]. According to the preference of fungi for water, *Fusarium* mycotoxins (ZEA, DON, and FUM) are more likely to be found in feed ingredients [17]. However, some studies present different findings. Complete feeds are highly contaminated with *Fusarium* mycotoxins compared with the feed ingredients. This finding could be because complete feeds are prepared with ingredients that contain a high amount of *Fusarium* mycotoxins [23].

The levels of mycotoxins vary substantially depending on the type of feed [16]. AFL often exceeds the limit in corn or cottonseed [1]. Contamination with AFL occurs in corn and peanut cake, which are also cocontaminated with FUM [11,22]. Wu *et al*. [13] identified contamination of distillers dried grains, which is a popular animal feed ingredient with AFL, ZEA, and DON. There were no significant differences observed between the types of samples in these studies.

These results suggest that mycotoxin contamination in feedstuffs is problematic. The problems increase if the feed contains various mycotoxins [24]. Besides the animal health risks, humans are impacted by the consumption of animals that have consumed contaminated feedstuff [3,6]. The permitted levels in animal feeds are a measure to control contamination. It is useful for producers to avoid animal health risks. However, the limits vary from country to country [1,12,25]. Thailand specifies only the AFL level permitted in animal feeds, because of the severity of the toxin [18]. As a consequence of the mycotoxin contaminations in the present study, other permissible limits should also be legally indicated [3,26]. The present study is limited because the data were collected from available records. Therefore, the association analyses did not deal with all possible factors.

## Conclusion

This study analyzed the prevalence of several mycotoxins from 2015 to 2020. High ZEA contaminations were evident in animal feeds. Contamination of 20-30% of samples with AFL and FUM was detected, whereas T-2 and DON contamination occurred in <10% of samples. Mycotoxin contamination in feedstuffs is, therefore, significantly related to the type of feed or ingredient. Our findings are important for producers to ensure that they can select and use animal feeds that reduce risks to animal health.

## Authors’ Contributions

SK carried out the statistical analysis and drafted the manuscript. PJ, SL, and AB prepared and arranged the data. AS reviewed and edited the manuscript. All authors have read and approved the manuscript.
